# The HO-1-expressing bone mesenchymal stem cells protects intestine from ischemia and reperfusion injury

**DOI:** 10.1186/s12876-019-1042-9

**Published:** 2019-07-12

**Authors:** Xue-Tao Yan, Xiao-Li Cheng, Xiang-Hu He, Wen-Zhong Zheng, Yuan Xiao-Fang, Chen Hu

**Affiliations:** 1 Department of Anesthesiology, Baoan Maternal and Child Health Hospital, Jinan University, Shenzhen, 518102 China; 2 Department of Anesthesiology, Zhongnan Hospital of Wuhan University, Wuhan, 430071 Hubei China

**Keywords:** Bone mesenchymal stem cells, HO-1, Intestinal ischemia and reperfusion injury

## Abstract

**Background:**

Bone mesenchymal stromal cells (BMSC) showed protective potential against intestinal ischemia. Oxygenase-1(HO-1) could alleviate oxidative stress. In the present study, we constructed HO-1-expressing BMSC and detected the effects of it on survival, intestinal injury and inflammation following intestinal ischemia and reperfusion injury (I/R).

**Methods:**

In this experiment, eighty adult male mice were divided into Sham, I/R, I/R + BMSC, I/R + BMSC/HO-1 groups. Mice were anesthetized and intestinal I/R model were established by temporarily occluding the superior mesenteric artery for 60 min with a non-crushing clamp. Following ischemia, the clamp was removed and the intestines were allowed for reperfusion. Prior to abdominal closure, BMSC/ HO-1 (2 × 10^6^ cells) or BMSC (2 × 10^6^ cells) were injected into the peritoneum of I/R mice respectively. Mice were allowed to recover for 24 h and then survival rate, intestinal injury and inflammation were determined. Reactive oxygen species (ROS) was assayed by fluorescent probe. TNFα and IL-6 were assayed by ELISA.

**Results:**

BMSC/HO-1 increased seven day survival rate, improved intestinal injury and down-regulated inflammation after intestinal I/R when compared with sole BMSC (*p* < 0.05 respectively). Multiple pro-inflammatory media were also decreased following application of BMSC/HO-1, when compared with sole BMSC (*p* < 0.05) respectively, suggesting that BMSC /HO-1 had a better protection to intestinal I/R than BMSC therapy.

**Conclusion:**

Administration of BMSC/HO-1 following intestinal I/R, significantly improved intestinal I/R by limiting intestinal damage and inflammation.

## Background

Ischemia reperfusion injury (I/R) is an important disease entity with a high mortality, disability, and mortality in the world. I/R are defined as injury caused by the restoration of blood supply after a period of ischemia, and it is the main cause of fatal intestinal diseases. Although oxygen levels are restored upon reperfusion, a surge in the generation of oxygen free radical (ROS) occurs and pro-inflammatory neutrophils infiltrate ischemic tissues to exacerbate ischemic injury and this damage is the direct reason of intestinal I/R. Despite the recent development in the treatment of intestinal I/R, mortality remains to be 50% or so [1]. During the development of intestinal ischemia, the sudden drop in intestinal blood flow may cause intestine necrosis, sepsis, multiple organ dysfunction, and ultimately death if not discovered early [2]. Intestinal I/R occurs in reperfusion injury of intestinal tissue, which has been proved to play an important role in the occurrence and progression of fatal infections and severe traumatic shock [3].

Mesenchymal stem cell (MSC) has the pluripotent, immunomodulatory, and proliferative potentials that help the wound repair of intestine tissues [4, 5]. They have the potential to differentiate into all kinds of cells [6, 7], showing antioxidant and anti-inflammation properties, enhancing tissue repair and alleviating intestinal I/R [8, 9]. Reperfusion of ischemic tissue activates a complex inflammatory response which leads to activation of innate and adaptive immune responses and contributes to injury, including activation of pattern-recognition receptors such as TLRs and inflammatory cell trafficking into the injured organ. Bone marrow-derived MSC (BMSC) has proved to down regulate the secretion of inflammatory mediators and oxidative injuries and improving intestinal I/R by decreasing intestinal permeability, villus injury and restoring the gut-mucosal barrier following injury [10, 11]. Although MSC from other tissue sources also showed similar protection against intestinal I/R, BMSC may have different protective effects according to current literatures, and BMSC is verified to have good protective effects, wide source and less carcinogenicity [10, 11]. Therefore, in this study, we chose this kind of stem cells.

Heme oxygenase-1 (HO-1), also known as heat shock protein, cleaves the heme and produces biliverdin, carbon monoxide and iron [12-14]. Many recent studies have established that HO-1 is induced by oxidative stress which is protective to tissue injury caused by I/R. Considering that some research has been done on the protective effects of BMSC on intestinal I/R, here we constructed a HO-1-overexpressing BMSC cell line and aimed to verify whether BMSC/HO-1 had stronger protective effects than sole BMSC on intestinal histological structure and inflammation after intestinal I/R.

## Methods

### Ethics statement

Male adult CD57BL/6 mice (25–30 g) obtained from Zhongnan Hospital of Wuhan University were maintained under specific pathogen-free condition. All experiments referring to the use of animals were approved by the Committee of Animal Care and Use of Bao’an Maternity and Child Health Hospital, China.

### CD57BL/6 mice BMSC

CD57BL/6 mice BMSC were purchased from sciencell, USA. The cells were expressing CD34 (−), CD44 (+), CD90 (+) and CD45 (−) identified by flow cytometry The culture medium and subculture kits for mice BMSC were purchased from Cyagen Bioscience, Inc. Briefly, the BMSC culture medium was made of CD57BL/6 mice BMSC basal medium supplemented with 10% BMSC-qualified fetal bovine serum and 1% penicillin-streptomycin.

### HO-1 over-expressing lentiviral vectors construction

Mice BMSCs with 10 or so passages were used for HO-1 over-expressing construction. Lentivirus carrying EGFP (vector) was supplied by Inovogen Tech. Inc., Beijing China. Primers were designed with reference to the HO-1 sequence of GenBank, 5′-TTATGAATTCATGGAGCGTCCACAGCCC-3′ (EcoRI site); and 5′-CGCGGGATCCTTACATGGCTAAATTCCCACTGC-3′ (BamHI site) and synthesized by Gene Pharma company (Shanghai, China). In this case, 2 μL cDNA was added into 50 μL total volume, and the reaction conditions are 94 °C 4 min, then 35 cycles of 94 °C 30 s, 55 °C 30 s and 72 °C 2 min, at last 72 °C 10 min. PCR product was digested by EcoRI+ BamHI double enzymes recovered from the gel by QIAquick Gel Extraction Kit. Then the PCR products were ligased with pEGFP-N1 and transformed into *E. coli* DH5α. A small quantity of plasmids was extracted by QIAprep Spin Miniprep Kit and sequenced by ABI377 DNA sequencer (Takara, Shanghai, China). BMSC were cultured in a 6-well plate (Corning, Inc., NY). After 24 h, we changed the culture medium, added pLV-EGFP-C or pLV-EGFP- HO-1, and maintained the cells in a humidified incubator with 5% CO_2_ at 37 °C. After 24 h, the old medium was replaced by fresh culture medium. Then the cultures were performed by medium change every 2–3 d. The transduction efficiency of the lentiviral vectors at 72 h after transfection was identified using fluorescence microscopy (Olympus Co., Tokyo, Japan).

### Real-time PCR

HO-1 over-expressing BMSC were obtained and cultured in cell culture medium. Total RNA was isolated from pLV-EGFP-C or pLV-EGFP- HO-1transfected cells using TRIzol reagent (Takara, Shanghai, China) according to the manufacturer’s instruction. RNA was reverse transcribed to cDNA using cDNA synthesis reagents (GenePharma, Shanghai, China). Primers used for the real-time PCR was synthesized by GenePharma (Shanghai, China): GADPH were 5′-ATGTTCAACTATTGGTGCTGGCA − 3′ and 5′-GGAGTTTCATTAGGTCCCGTTTGT -3′; HO-1 were 5′- TGACAGAAGAGGCTAAGACCGC-3′ and 5′-AGAGTGAGGACCCACTGGAGGA-3′. The expression level was analyzed using 2^-ΔΔCt^ Method [15].

### Western blotting

pLV-EGFP-C or pLV-EGFP-HO-1 transfected cells were collected and total cellular proteins from were extracted and separated on SDS-PAGE gels (12%). Then, proteins were incubated with primary rabbit polyclonal antibodies to HO-1 (1: 250 dilutions, Abcam Ltd., Cambridge, UK) or GAPDH (1: 500 dilution, Abcam Ltd., Cambridge, UK). After washing three times with phosphate buffer saline (PBS) containing 0.1% Tween 20 and then incubated with goat anti-rabbit IgG conjugated with horseradish peroxidase (1:2000, Boster, Wuhan, China). Immunoreactive complexes were visualized using chemiluminescence reagents (Thermo Scientific, USA) [16].

### Murine intestinal I/R model

CD57BL/6 mice (*n* = 80) obtained from Zhongnan Hospital of Wuhan University Bao’an Maternity and Child Health Hospital, China. The animals were kept in cages with 12 h light-dark cycle. CD57BL/6 mice were divided into 4 groups: sham group, I/R group, IR + BMSC group and I/R + BMSC/HO-1 group**.** Ten mice per group were used for 7-day survival analysis and 10 mice per group for other assays at 24 h after reperfusion.

After intraperitoneal injection of 2% pentobarbital sodium, the animals were fixed on a heating pad to maintain their body temperature and the hair in the abdominal was removed. After routine disinfection of the abdomen region, the median incision was inserted into the abdomen to separate the superior mesenteric artery (SMA). In the sham group, the same operation was performed, and the abdomen was kept open for the same time as in the I/R group. But other groups were clipped to the root of SMA with microartery clips. After 60 min occlusion of SMA, the blood supply was restored and the incision was closed. Then 200 μL PBS, BMSC (10^7^cells/ml), or BMSC /HO-1 (10^7^cells/ml) were injected into the intraperitoneal cavity of various groups respectively. After wound closure, antibiotic ointment was applied to the incision and buprenorphine (1 mg/kg) and caprofen (5 mg/kg) were injected into the intraperitoneal cavity. Mice were then allowed to awaken from anesthesia.

### Survival analysis

After I/R, the death time of experimental mice were recorded daily. Mice in various groups were monitored for 7 d and survival curves were plotted. Remaining mice were injected isoflurane overdose and given cervical dislocation at end point of the 7 d.

### Small intestinal injury evaluation by HE staining

After 24 h of reperfusion, mice in each group were executed and terminal ileums were harvested at 24 h after I/R and fixed in 4% paraformaldehyde with subsequent dehydration in 70% ethanol. Paraffin-embedded sections were prepared and stained with HE staining. The degree of small intestinal injury was evaluated according to the article by Ceulemans et al. [17] as following 0, normal villus and gland; 1, the top of villi epithelium with mild damage; 2, mild damage under epithelial glands; 3, expand the subepithelial space; 4, moderate separation of epithelium from lamina propria and impaired gland; 5, the top of the part of the villi fell off; 6, dilatation of the villi and capillary dilatation; 7, the villi of the lamina propria fall off, and the gland is damaged obviously; 8, digestion and decomposition of the lamina propria; 9, bleeding and ulcers. Three consecutive slides from each sample and 5 fields of each slide with a magnification× 200 were observed by a professional pathologist, and the average scores of each group was calculated. The tests were random and double-blind.

### ROS assay by flow cytometry

Following euthanasia, mouse intestinal tissues were harvested and homogenized in ice-cold RIPA buffer with protease and phosphatase cocktail inhibitors (1:100; Sigma, MO, USA). The proteins were obtained at 10,000 rpm for 30 min and the protein concentration was quantified by BCA kit (Abnova, CA, USA). ROS production was determined using a ROS assay kit (Beyotime Biotech, Shanghai, China) by incubation with 10 μmol/L DCFH-DA at 37 °C for 30 min in the dark. The ROS level was detected using a FACSCalibur flow cytometer (BD Biosciences, NJ, USA).

### Elisa

Interleukin-6 (IL-6) and TNF-α in intestinal tissues and serum were quantified with ELISA kits (Neobioscience, Shanghai, China) according to the manufacturer’s instructions and are reported as nanograms cytokine per gram of total intestinal protein.

### Statistical analysis

All statistical analyses were performed with SPSS 15.0 to assess differences among the groups. Small intestinal injury score evaluation was completed by one-way ANOVA within multiple groups and Bonferroni post-hoc analysis between two groups. Other assays were carried out by one-way ANOVA within multiple groups and by least significant difference (LSD) between two groups. Differences with a *p* value less than 0.05 were considered significant.

## Results

### Identification of HO-1 over-expressing vector

**Fig. 1:**
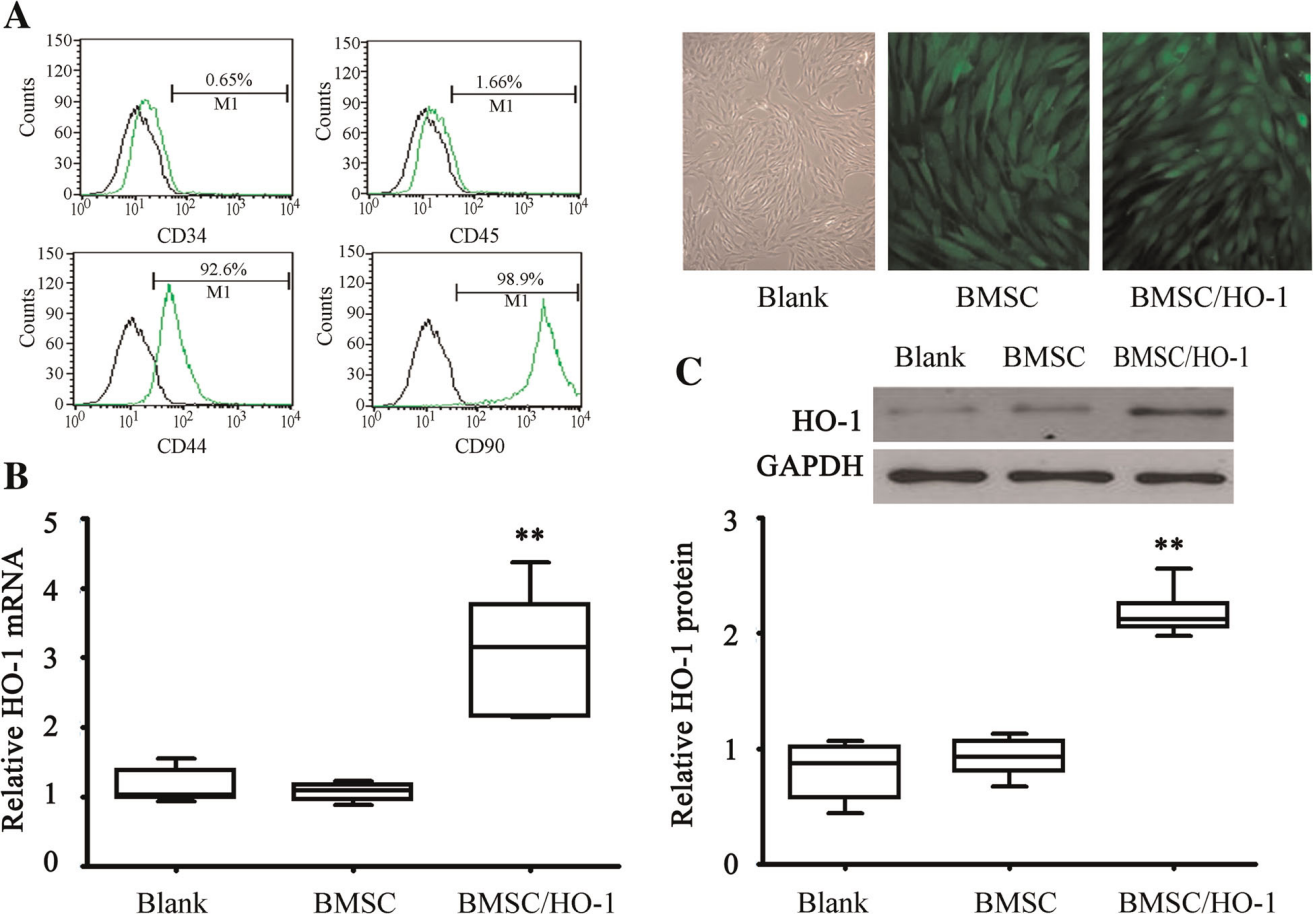
Identification of lentiviral vectors-mediated HO-1 expression in BMSC. (**a**) The transduction efficiency of lentivirus was assayed using fluorescence microscopy, × 400; (**b**) The mRNA expression of HO-1 was assayed by real-time PCR (*n* = 4); (**c**) The HO-1 protein expression was assayed by Western blot (n = 4).* *p* < 0.05 vs. BMSC

The successful transfection was detected by fluorescence microscopy for the expression of GFP. As shown in Fig. 1a, after transfection of lentiviral vector with a multiplicity of infection (MOI) of 100 (ratio of virus to cell number), the transduction efficiency was above 90%. Moreover, after transfection of BMSC/HO-1, the GFP in the nucleus was higher than that in the empty vector group. We next detected the HO-1 mRNA expression in the transfected BMSC and the results showed that HO-1 mRNA levels were significantly higher in the HO-1 group than that in the empty vector group (* *p* < 0.05, Fig. 1b). The results from western blotting showed that the HO-1 protein expression in the cells was also increased significantly in the HO-1 group (Fig.1c), compared with the empty vector group (* p < 0.05). These results suggested that HO-1 gene expression mediated by lentiviral vectors is efficient and relatively stable.

### BMSC/HO-1 improved survival rate after intestinal I/R

**Fig. 2:**
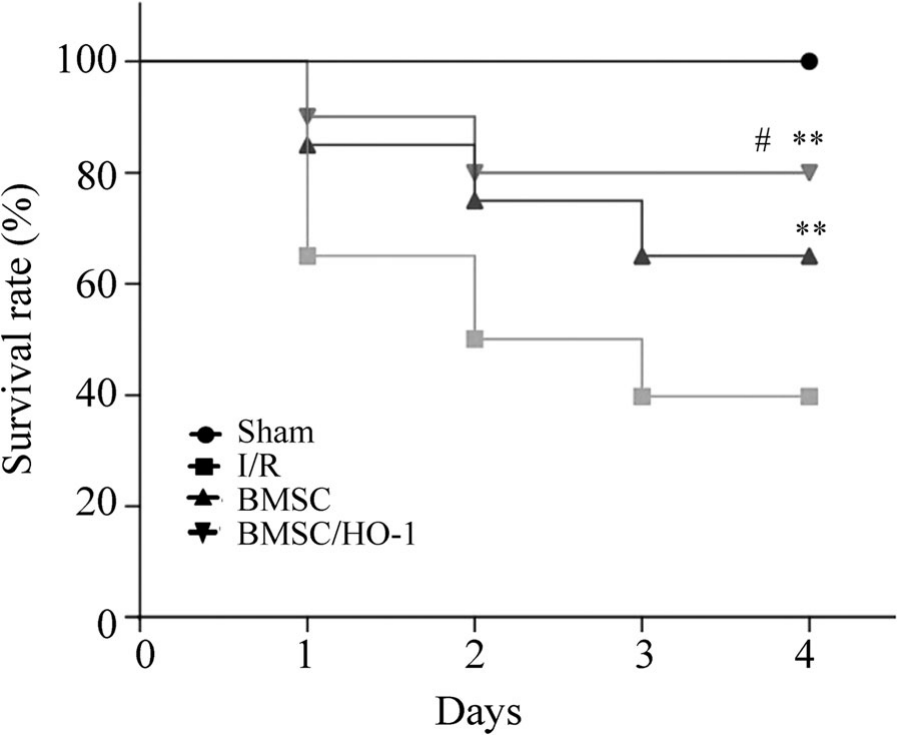
BMSC/HO-1 improved 7 day survival rate (*n* = 10). Seven day survival rate in BMSC/HO-1 or BMSC groups was significantly improved when compared with that in I/R group. Also, there had statistically significant differences between BMSC/HO-1group and BMSC groups. .* *p* < 0.05 and ** *p* < 0.01 vs. I/R; # *p* < 0.05 vs. BMSC

Survival curve was obtained from mice model following I/R injury (*n* = 10, Fig. 2) and death rate of I/R mice at 24 h was 40%. The results also showed that in BMSC/HO-1 or BMSC groups, seven day survival rates from Day 3 were 80 and 62% respectively which had significantly difference with that in I/R group (40%, ** *p* < 0.01). There had statistically significant survival from Day 3 in BMSC/HO-1group when compared with BMSC groups.

### BMSC/HO-1 improved histological architecture

**Fig. 3:**
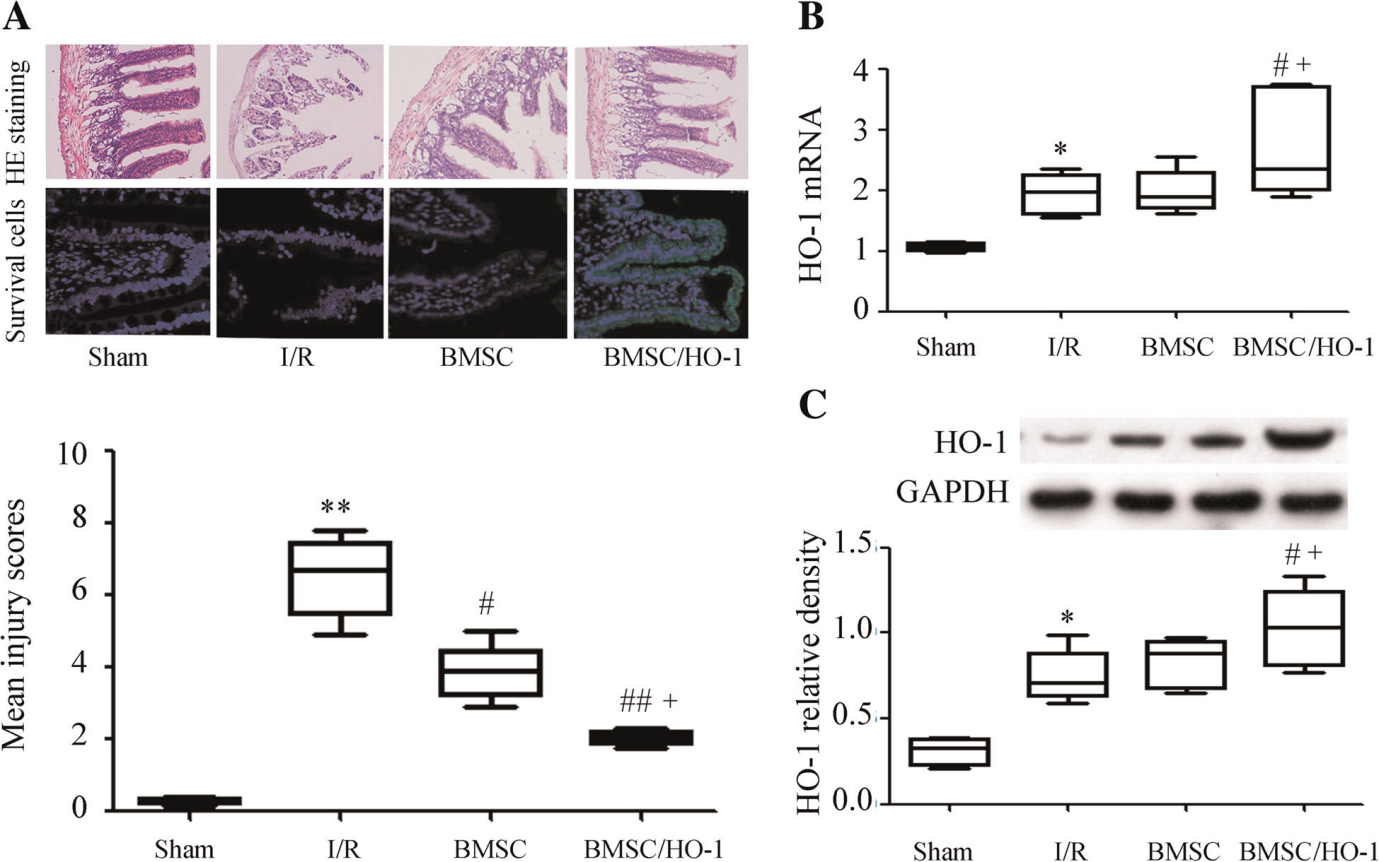
BMSC/HO-1 improved histological architecture. **a**, HE staining and transplanted cell detection of small intestine following intestinal I/R and BMSC/HO-1 or BMSC treatment (HE, × 200). **b**, The mRNA expression of HO-1 was assayed by real-time PCR (*n* = 6); **c**, the HO-1 protein expression was assayed by Western blot (n = 6). ** *p* < 0.01 vs. Sham; # *p* < 0.05 and ## *p* < 0.01 vs. I/R; + *p* < 0.05 vs. BMSC

There was no obvious abnormal change of small intestine tissue in the Sham operation group. Intestinal injury after I/R was severe, characterized by a large amount of villus damage, a small amount of epithelial necrosis, detachment, the top of the intestinal Villi collapse, subendothelial hemorrhage and neutrophils diffusion. As expected, intestinal histological architecture following I/R injury at 24 h of reperfusion was significantly improved by the use of BMSC or BMSC/HO-1 (Fig. 3a). By tracing the transplanted cells, we found that more transplanted cells survived in BMSC/HO-1 group than those in BMSC group (Fig. 3a). After 24 h reperfusion, mean injury scores were also significantly improved in both BMSC treated groups (BMSC (3.147 ± 0.25462, *p* < 0.05), BMSC/HO-1 (1.5367 ± 0.1634, *p* < 0.01) compared to that in I/R group (6.52 ± 0.4657). Moreover, there had significant difference between BMSC/HO-1 and BMSC. These data indicate that BMSC/HO-1 had a better protection against intestinal I/R injury than BMSC.

We next detected the HO-1 mRNA expression in intestine tissues and the results showed that HO-1 mRNA levels were significantly higher in the BMSC/HO-1 group than that in I/R group or BMSC group (*n* = 6, *p* < 0.05, Fig. 3b). The results from western blotting showed that the HO-1 protein expression was also increased significantly in the BMSC/HO-1 group (*n* = 6, Fig. 3c), compared with that in the I/R group or BMSC group (*p* < 0.05).

### BMSC/HO-1 attenuated production of ROS, IL-6 and TNF-α

**Fig. 4:**
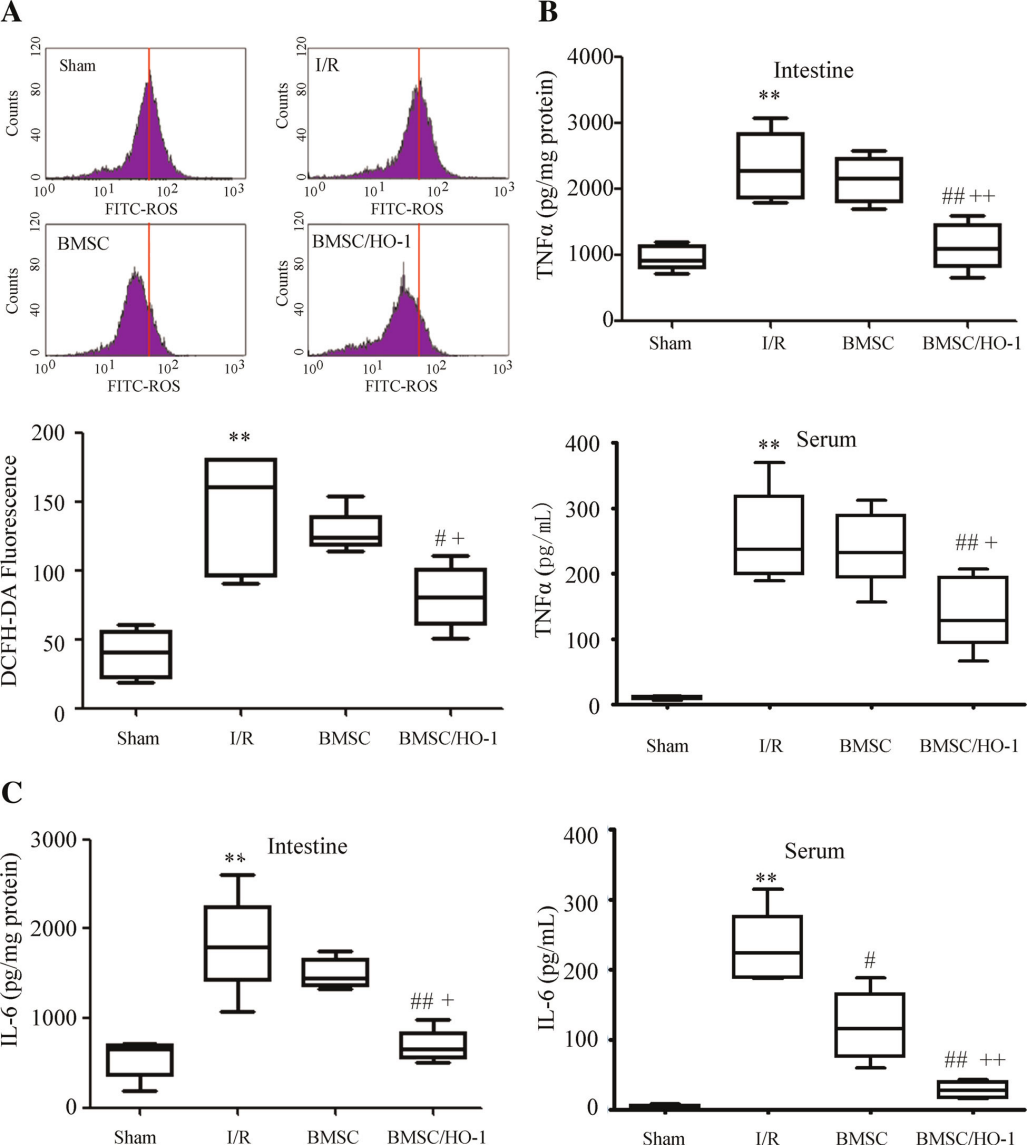
BMSC/HO-1 attenuated production of ROS, IL-6 and TNF-α (*n* = 6). **a** ROS production; **b** TNF-α production; **c** IL-6 production in intestine and serum. Levels of ROS in intestine at 24 h of reperfusion were significantly higher than those in sham group and decreased after treatment of BMSC/HO-1. Moreover, IL-6 and TNF-α were significantly lower in BMSC/HO-1 group at 24 h compared to those in I/R group. ** *p* < 0.01 vs. Sham; # *p* < 0.05 and ## *p* < 0.01 vs. I/R; + *p* < 0.05 and ++ *p* < 0.01 vs. BMSC

As shown in Fig. 4, ROS content in the intestinal tissue of I/R was significantly higher than that of sham (*n* = 6, *p* < 0.01). Compared with that in I/R group, ROS content in intestinal tissue of BMSC/HO-1 group decreased significantly (n = 6, p < 0.05), while ROS content in BMSC group had no significant difference. Moreover, the levels of IL-6 and TNF-α protein in intestinal tissues and serum of I/R group were significantly higher than those of sham group (n = 6, *p* < 0.01). Compared with those in I/R group, IL-6 and TNF-α secretion had no significant differences in BMSC group, while significantly decreased in BMSC/HO-1 group (n = 6, *p* < 0.01).

## Discussion

The intestinal tract is one of the most sensitive tissues to ischemia. The major arterial blood supply of intestine is superior and inferior mesenteric arteries. The blood supply from these two major arteries overlap, with abundant collateral circulation. However, there are weak points. The mucosa of intestine is the layer of most sensitive to hypoxia because of its high metabolic activity. Intestinal I/R are common in trauma, shock, intestinal obstruction, abdominal surgery, etc. [18]. BMSC is a kind of adult stem cells with the potential of multiple-direction differentiations, which can be differentiated into specific tissue cells induced by local microenvironment when they are injured, and can participate in repair.

Intestinal injury often causes systemic inflammatory response syndrome and multiple organ dysfunction syndromes which is the one of main reasons of high morbidity and mortality. Various inflammatory cells including mucosal cells, endothelial cells, macrophage, neutrophils, fibroblast are responsible for secretion of various cytokines, chemokines, free radicals following ischemia. It is believed that the mechanism of injury is related to the release of ROS after hypoxia [19, 20]. Therefore, it is a strategy to treat intestinal I/R by inhibiting oxidative stress.

HO-1, a 32 kDa enzyme containing 288 amino acid residues, is a stress-induced isoform present throughout the body. HO-1 has been reported in the mitochondria, cell nucleus, and plasma membrane [21]. HO-1 can attenuate oxidative damage and protect the myocardial, neurological and renal I/R injury in previous research and the expression of HO-1 in response to different inflammatory mediators may contribute to the resolution of inflammation and has protective effects against oxidative injury [22, 23].

In this study, we found a distinct survival advantage with the use of BMSC or BMSC/HO-1. Moreover, BMSC/HO-1 had more significant improvements in survival rate (80%), and histological injury scores as compared to BMSC. Activation of mononuclear and macrophage cells in the process of injury release large amounts of TNF-α and interleukins, causing a cascade reaction and a large release of the inflammatory media. The gastrointestinal tract is the main target of TNF-α. Intestinal I/R causes a significant increase in the expression of TNF-α [24]. In this study, the contents of TNF-α and IL-6 in the intestine and serum of I/R group increased significantly, and the release of TNF-and IL-6 decreased significantly after administration of BMSC/HO-1, indicating that BMSC/HO-1 may function more effectively to limit inflammation compared to BMSC.

There are several limitations in the present study. The assessment of inflammatory media may have a wide variation among different groups. Moreover, multiple mechanisms for BMSC/HO-1 protective effects may exist. Therefore, further studies that label and track BMSC/HO-1 after transplantation may yield further insight into the protective mechanism of BMSC/HO-1.

## Conclusion

In conclusion, BMSC/HO-1 therapy is an effective option for intestinal I/R. BMSC/HO-1 improved survival, intestinal injury and inhibited inflammation, which had a more profound effect on reducing tissue inflammation than BMSC.

## Data Availability

All data are presented within the manuscript.

## Abbreviations

BMSCs: Bone mesenchymal stromal cells
HO-1: Heme oxygenase-1
I/R: ischemia and reperfusion injury
MSCs: Mesenchymal stem cells
SMA: the superior mesenteric artery
